# Long-Term Effectiveness and Cost-Effectiveness of Metformin Combined with Liraglutide or Exenatide for Type 2 Diabetes Mellitus Based on the CORE Diabetes Model Study

**DOI:** 10.1371/journal.pone.0156393

**Published:** 2016-06-15

**Authors:** Xuesong Zhang, Sisi Liu, Yukun Li, Yan Wang, Meimei Tian, Guoqiang Liu

**Affiliations:** 1 CT Department, the Third Hospital of Hebei Medical University, Shijiazhuang, China; 2 Department of Pharmacy, the Third Hospital of Hebei Medical University, Shijiazhuang, China; 3 Second Department of Endocrinology, the Third Hospital of Hebei Medical University, Shijiazhuang, China; Weill Cornell Medical College Qatar, QATAR

## Abstract

**Background:**

Type 2 diabetes mellitus (T2DM) is associated with β cell impairment. Agonists of the glucagon-like peptide 1 (GLP-1) receptor (such as liraglutide and exenatide) can increase the number of pancreatic β cells and improve cell function. These drugs contribute to the long-term control of T2DM.

**Objective:**

To evaluate the cost-effectiveness of metformin combined with liraglutide or exenatide in Chinese patient with T2DM.

**Methods:**

Patients with T2DM from the Third Hospital of Hebei Medical University were treated with oral metformin combined with liraglutide (0.6 mg/day, could be increased by 0.6 mg weekly until 1.2 or 1.8 mg) or exenatide (5 μg bid for four weeks, increased to 10 μg bid). The computer simulation model CORE was used to calculate the 30-year expected life expectancy, quality-adjusted life years (QALY), direct costs, HbA1c levels, body mass index (BMI), and the incidence of cardiovascular, renal, and ocular complications of T2DM. Patients were followed up for 52 weeks. Medication costs were calculated according to retail prices in China. A 3% annual discount was adopted in this study.

**Results:**

The 30-year simulation showed that the total direct medical costs were lower using liraglutide compared to exenatide by 2130 RMB/QALY yearly, while the expected life expectancy and QALY were increased by 0.471 years and 0.388, respectively, using liraglutide with an incremental cost-effectiveness of -11,550 RMB/QALYs.

**Conclusion:**

Liraglutide 1.2 mg/day was superior to exenatide 10 μg bid with respect to cost-effectiveness in Chinese patients with T2DM.

## Introduction

In China, 92.4 million Chinese adults are with T2DM and 148.2 million Chinese adults are with prediabetes [1]. In Chinese patients, failure of β-cell function might be one of the main reasons for pre-diabetes developing into T2DM instead of aggravated insulin resistance, as in Western populatifigns [2]. Therefore, protecting the function of the β cells is an important treatment strategy for the long-term control of T2DM in China.

The glucagon-like peptide 1 (GLP-1) is secreted by L cells of the terminal ileum and colon after food intake under physiological conditions [3] and its half-life is only of 1–2 minutes [4]. Therefore, GLP-1 receptor agonists such as exenatide and liraglutide were engineered to increase the drug action time [5]. GLP-1 agonists are widely accepted for T2DM treatment, but are still regarded as second line medication by the ADA guidelines [6,7]. Liraglutide has been shown to improve glycemic control in patients with T2DM and to have a low frequency of adverse effects [8,9]. Its effect has been shown to be similar in Asian patients compared to the general population [10]. A meta-analysis of exenatide, insulin, and pioglitazone showed that exenatide was the most potent of the three drugs for glycemic control and improving β cell function [11].

The CORE Diabetes Model (CDM) can be used to project the long-term clinical and economic outcomes associated with liraglutide treatment for T2DM within the USA setting. The structure and validation of this model have been described in details [12,13]. The CDM is a validated, non-product-specific policy analysis tool that performs real-time simulations, taking into account specific diabetes treatments [12,13]. The development of diabetes and its complications, clinical treatment, therapeutic outcomes, resource utilization, and costs can be simulated in the CORE model, which uses the Markov model, in which the long-term therapeutic effect and cost are predicted by calculating the switching ratio of different Markov status in a certain period [12,13].

Therefore, this study aimed to determine quality-adjusted life years (QALY) based on utility value of diabetes and the damage caused by disease-related events, which were derived from the published research results [13,14]. A 3% discount rate was adopted in study for CORE diabetes model as international default, to simulate the long-term therapeutic outcomes and costs in 30 years for patients with T2DM in China. The analysis was based on a follow-up period of 52 weeks. The results of this study could provide some clues for clinicians when selecting the most appropriate treatment for Chinese patients with T2DM.

## Material and Methods

### Patients

Data were collected from patients with T2DM and newly prescribed with exenatide or liraglutide, and who visited the Third Hospital of Hebei Medical University between November 2011 and March 2013. This observational and non-interventional study was carried out over a period of 52 weeks. The patients were treated with either exenatide or liraglutide combined to metformin, lipid-lowering drugs, and/or antihypertensive drugs.

The inclusion criteria were: 1) patients fulfilling the diagnostic criteria of the “Chinese type 2 diabetes treatment and prevention guidelines” issued in 2010; 2) aged 18–80; 3) metformin alone was not potent enough after a period of 3 months (HbA1c levels kept between 7% and 11%); 4) no previous treatment with a GLP-1 agonist; 5) body mass index (BMI) ≥24 kg/m^2^ [10]; 6) for patient with hypertension, blood pressure had to be controlled for at least 1 month. Exclusive criteria were: 1) severe cardiovascular or liver or kidney diseases; 2) diabetic ketosis; 3) endocrine tumor or inflammatory disorder; and 4) infectious or gastrointestinal diseases.

This study was approved by the ethics committee of the Third Hospital of Hebei Medical University (L2011-001-1). All patients provided a written informed consent.

### Treatment regimens

Liraglutide (Novo Nordisk, Bagsvaerd, Denmark) was subcutaneously injected at an initial dose of 0.6 mg once a day at fixed time. The dose could be increased by 0.6 mg weekly until 1.2 or 1.8 mg subcutaneously once a day if there was no intolerance such as nausea or vomiting. Exenatide (Eli Lilly, Indianapolis, IN, USA) was subcutaneously injected at an initial dose of 5 μg twice a day (before breakfast and dinner) for four weeks, and increased to 10 μg twice a day. Other medications (metformin, angiotensin converting enzyme inhibitors (ACEI), angiotensin receptor blockers (ARB), acetylsalicylic acid, statins, and/or fibrates) were taken according to the prescriptions of the treating physicians without restriction to makers.

### Database input

The CORE Diabetes Model (CDM), a validated non-product-specific policy analysis tool, were used to analysis the cost-effectiveness of two treatment protocol for T2DM [12,13]. Treatment-related information was input into the model including therapeutic strategies, age, gender, baseline risk factors, screening test results, complications, adverse effects, therapeutic costs, costs for complications, other medication, and laboratory tests. This model was used to simulate the long-term therapeutic outcomes.

### Cost input

The direct costs were calculated from the perspective of society including medication costs, diagnostic costs, adjuvant examination costs, nursing costs, rehabilitation costs, and self-monitoring blood glucose costs. The none-measurable costs such as depreciation charge for medical equipment, indirect medical costs, disease onset and death costs, and other intangible costs including pain and sadness were supplemented by the model. The highest retail price issued by the Provincial Price Bureau and the Reform Commission since 2013 was adopted. The frequency and costs of self-monitoring of blood glucose was recommended according to the Guidelines for diabetes care and education in China [15]. All cost-related data were adjusted to the levels of 2012 according to the Chinese consumer price index (CPI).

### Sensitivity analysis

Given the chronic process of T2DM, the observation on the clinical effect and cost of the patients were discounted, which might cause some significant influence due to the varied period and discount rates. In this study, the analysis was conducted for long-term health outcomes and the cumulative rate of complications with the period preset to 40 and 50 years, and discount rate preset to 0% and 5% to further evaluate the robustness of the long-term results.

### Measurements

Life expectancy is the expected life span of one specific subject. QALY refers to the years after life quality adjusted; for instance, compared with healthy people, the quality life of patients is less due to diseases or disabilities. Direct costs are the direct costs of clinical management including medication, diagnosis, adjuvant examination, nursing, rehabilitation, and self-monitoring of blood glucose. HbA1c is used to reflect the blood glucose control within the prior 8–12 weeks. BMI is calculated by dividing the body weight (in kg) divided by squared height (in m). The incremental cost effectiveness ratio is the effect difference divided by the cost difference. A bootstrap scatter diagram is the scatter diagram of incremental costs and incremental effects plotted by 1000 simulations using various averaged measurements of T2DM through the non-parametric Bootstrap method (CORE model) [16].

### Statistical analysis

SPSS 13.0 (SPSS Inc., Chicago, IL, USA) was used for statistical analysis. The normality of the continuous data was tested using the Shapiro-Wilk test. Normally distributed data are presented as mean ± standard deviation (mean ± SD). Non-normally distributed data are presented as median M (P25, P75). For the comparison between before and after treatment, paired *t* test or Wilcoxon rank test were used as appropriate. All statistical tests were two-sided, P>0.10 was considered significant for the normality test and P<0.05 was considered as statistically significant for the other tests.

## Results

### Evaluation measurements and baseline data

There were 113 and 92 patients who were newly prescribed liraglutide and exenatide, respectively, between November 2011 and March 2014. Among them, 35 patients stopped liraglutide (19 with gastrointestinal disorders, 10 for economic causes, and six were lost to follow-up), while 28 patients stopped exenatide (20 with gastrointestinal disorders, five for economic causes, and two were lost of follow-up). There were 68, 10, and 64 patients undergoing treatment with 1.2 mg of liraglutide, 1.8 mg of liraglutide, and 10 μg of exenatide, respectively. Because of the small number of patients in the 1.8 mg liraglutide group, the 1.2 mg liraglutide and 10 μg exenatide groups were finally selected for subsequent analyses. The detailed baseline data are shown in Table 1.

**Table 1:** Baseline characteristics of the patients.

Variables	Liraglutide	Exenatide	*P*
Baseline age (year)	49.6±12.5	54.1±9.5	NS
Disease course (year)	7.8±6.4	12.8±8.2	<0.05
Ratio of man (%)	55.1	59.3	NS
Smoking (%)	29.5	40.1	<0.05
HbA1c (%)	8.63±1.5	8.66±0.8	NS
BMI (kg/m^2^)	30.33±3.9	30.40±8.6	NS
Systolic pressure (mmHg)	135.30±17	135.00±15	NS
Total cholesterol (mmol·L^-1^)	5.0±1.1	4.1±0.6	NS
High density lipoprotein (mmol·L^-1^)	1.0±0.2	1.2±0.4	NS
Low density lipoprotein (mmol·L^-1^)	3.1±0.9	2.9±1.1	NS
Triglyceride (mmol·L^-1^)	3.0±2.2	2.8±1.9	NS

Data are expressed as mean ± SD. NS: no significant difference

### Therapeutic effects

HbA1c, BMI, systolic blood pressure, total cholesterol, and low-density lipoprotein were all decreased in both groups after 52 weeks of treatment (S1 Table). High-density lipoprotein levels were not improved in patients treated with exenatide. Triglyceride levels increased in patients treated with liraglutide. There was no hypoglycemia reported.

### Effect on HbA1c after long-term simulated treatment of liraglutide and exenatide

The simulated long-term HbA1c curve of patients with T2DM is shown in S1 Fig. Over 30 simulated years, the HbAc1 levels were slightly lower in patients treated with liraglutide compared to exenatide: 9.05% and 9.60%.

### Effect on BMI after long-term simulated treatment with liraglutide and exenatide

BMI was decreased in both groups after one year of treatment, with 28.91 kg/m^2^ for liraglutide and 29.26 kg/m^2^ for exenatide, and BMI remained unchanged afterwards (S2 Fig). There was no significant difference between two groups.

### Effect on life span after long-term simulated treatment with liraglutide and exenatide

**Fig 1:**
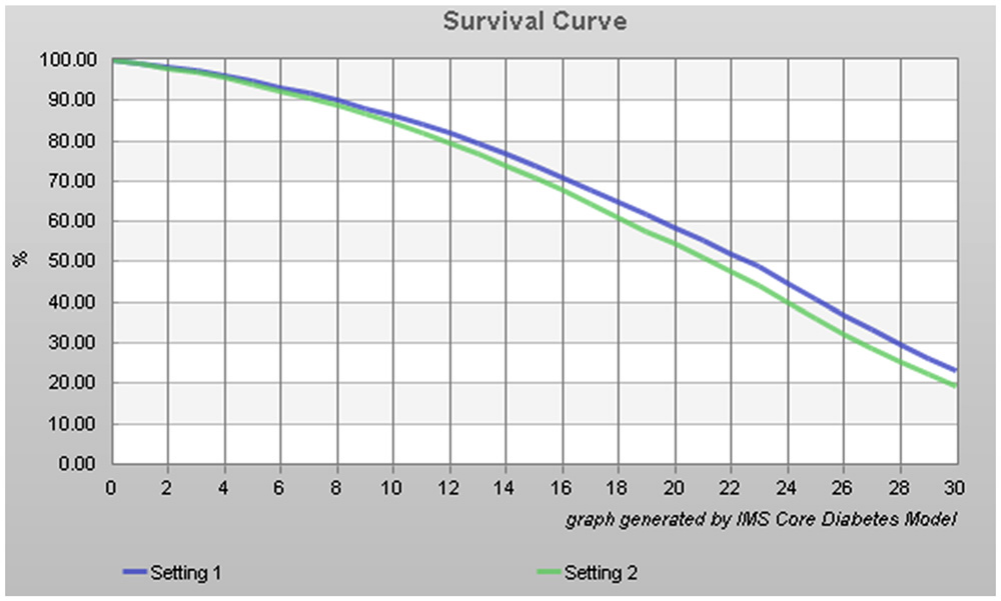
30-year simulated survival curve of diabetes patients. Blue is liraglutide and green is exenatide.

There were 23.2% and 19.4% of patients surviving after 30 years in the liraglutide and exenatide groups, respectively, as shown in Fig 1. Survival dropped below 50% after 23 and 22 years of treatment with liraglutide and exenatide treatment, respectively.

### Effect on diabetic complications after long-term simulated treatment with liraglutide and exenatide

The 30-year simulation suggested that the occurrence time of initial complication was relatively later under liraglutide treatment compared to exenatide (S2 Table). The incidence of complications except angina, myocardial infarction, and stroke were all higher under exenatide treatment compared with liraglutide (Table 2). Furthermore, the cumulative rates of complications were all higher under exenatide treatment compared with liraglutide with the period preset to 40 (S3 Table) and 50 (S4 Table) years. Moreover, the life expectancy and QALY were 0.471 years and 0.388 QALYs higher under liraglutide treatment than under exenatide (Table 3).

**Table 2:** The cumulative rate of diabetic complications with the period preset to 30 years.

Compilations	Liraglutide(%)	Exenatide(%)	Changes(%)
Eye			
Background retinopathy	27.312	26.358	0.954
Proliferative retinopathy	0.592	0.549	0.043
Severe visual impairment	12.160	11.554	0.606
Macular edema	26.465	25.515	0.950
Cataract	13.524	12.978	0.546
Kidney			
Microalbuminuria	26.980	25.872	1.108
Large amount of proteinuria	9.427	8.769	0.658
End stage renal disease	1.891	1.730	0.161
Kidney related death	1.462	1.348	0.114
Foot			
Foot ulcer (first)	40.996	39.296	1.700
Foot ulcer (repeated)	58.241	54.505	3.736
Amputation (first)	12.535	11.830	0.705
Amputation (multiple times)	4.779	4.422	0.357
Nervous system			
Neuropathy	69.411	67.326	2.085
Blood vessel			
Peripheral vascular disease	19.784	19.634	0.150
Congestive heart failure (disease)	21.964	21.831	0.133
Congestive heart failure (death)	9.814	9.657	0.157
Angina pectoris	21.344	24.783	-3.439
Myocardial infarction (onset)	30.651	38.299	-7.648
Myocardial infarction (death)	23.484	29.116	-5.632
Stroke (onset)	23.836	24.328	-0.492
Stroke (death)	12.581	12.829	-0.248
Mild hypoglycemia event	20.615	19.575	1.04
Lactic acidosis	15.954	15.153	0.801

**Table 3:** Long-term simulated health outcomes and costs.

Parameters	Liraglutide	Exenatide	Changes
Life expectancy (years)	14.506	14.035	0.471
Quality-adjusted life year (QALY)	10.018	9.630	0.388
Total costs (RMB)	407,582	412,065	-4483
Costs of treatment (RMB)	245,227	247,357	-2130
Management costs (RMB)	43,517	42,464	1053
Costs of treatment for cardiovascular diseases (RMB)	58,385	64,458	-6073
Costs of kidney disease (RMB)	2807	2677	130
Costs of treatment of ulcer, amputation, and neuropathy (RMB)	54,789	52,389	2400
Eye disease treatment costs (RMB)	2855	2720	135

### Effect on therapeutic costs after long-term simulated treatment with liraglutide and exenatide

The total cost of liraglutide treatment was 4,483 RMB less than for exenatide treatment, and the therapeutic cost as well as cardiovascular complication treatment cost of liraglutide treatment was 2,310 RMB and 6,073 RMB less than for exenatide treatment, respectively, but the management cost and the cost for the other complications were higher under liraglutide treatment. The cost for kidney complications was small than all other costs (Table 3).

The long-term simulation results (discount rate of 3% and 30 years) indicated that the total direct medical cost was lower with liraglutide, while the life expectancy and QALY were increased by 0.471 years and 0.388 QALY, respectively, with liralgutide compared to exenatide (Table 3). The incremental cost/effectiveness was -11,550 RMB/QALYs with liraglutide (Table 4, 3% discount rate at years 30, 40, and 50).

**Table 4:** Long-term health sensitivity analysis.

	Life expectancy (years)			Quality adjusted life year (QALY)			Costs (RMB)			ICER
Item	Liraglutide	Exenatide	Change	Liraglutide	Exenatide	Change	Liraglutide	Exenatide	Change	
Period										
30 years	14.506	14.035	0.471	10.018	9.630	0.388	245227	247357	-2130	-11550
40 years	14.869	14.313	0.556	10.221	9.779	0.442	424728	425876	-1148	-2597
50 years	14.899	14.369	0.53	10.235	9.812	0.423	426491	428060	-1569	-3711
Discount rate										
0%	20.456	19.62	0.836	13.873	13.218	0.655	582046	582248	-202	-308
5%	11.91	11.579	0.331	8.313	8.029	0.284	333277	339019	-5742	-20233

ICER, Incremental cost-effectiveness ratio.

**Fig 2:**
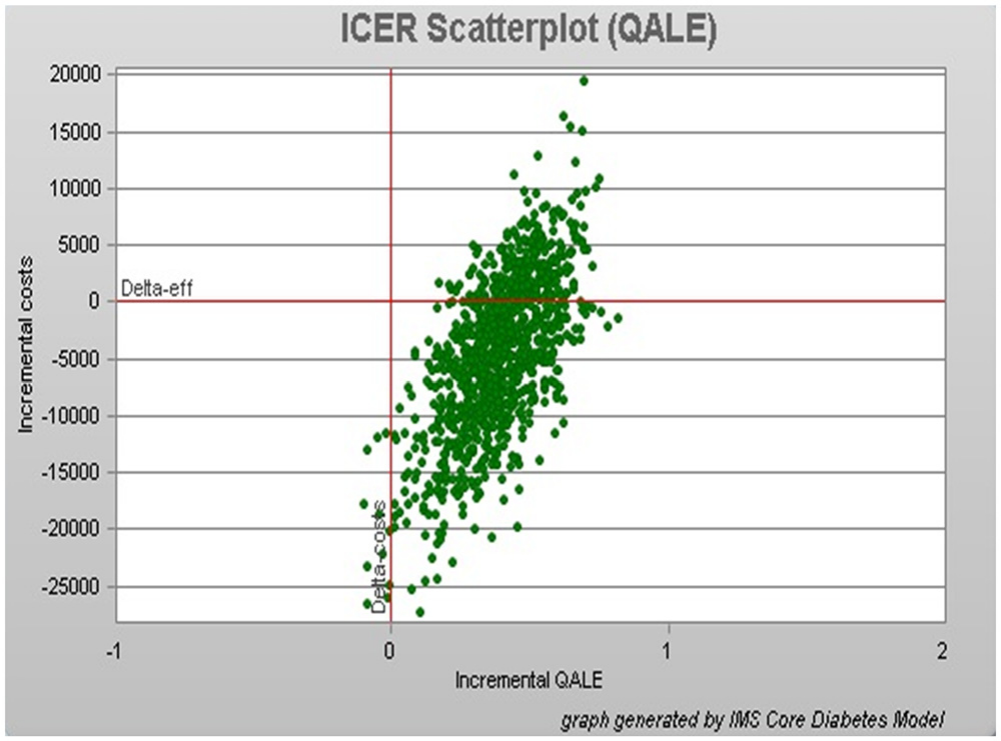
Base-case scatter plots generated for of 1000 type 2 diabetes patients treated with liraglutide vs. exenatide.

Bootstrap scattered plot dots were mainly concentrated in the right lower quadrant, the QALE was used as an incremental effect index (Fig 2).

### Sensitivity analysis for long-term simulated treatment with liraglutide and exenatide

The QALY was 10.221 and 10.235 for liraglutide treatment for simulated years 40 and 50 at a discount rate of 3%, which were longer than that of exenatide. Moreover, the direct cost of liraglutide treatment was lower than that of exenatide. When the simulated treatment period was set as 30 years with a discount rate of 0% and 5%, the liraglutide treatment was even more superior and economic(Table 4).

## Discussion

As a chronic disease requiring long-term medical care, the costs of T2DM treatment is an important concern for the society. In this study, the 30-year simulation showed that the total direct medical costs were lower using liraglutide compared to exenatide by 2130 RMB/QALY each year, while the expected life expectancy and QALY were increased by 0.471 years and 0.388, respectively, using liraglutide with an incremental cost effectiveness of -11,550 RMB/QALYs. The total cost of liraglutide treatment was 4,483 RMB less than with exenatide, and the therapeutic cost as well as cardiovascular complication treatment cost of liraglutide treatment was 2,310 RMB and 6,073 RMB less than with exenatide, respectively.

In this study, liraglutide combined to metformin decreased the mean HbA1c values by 1.1% after 52 weeks of treatment. In a previous study, the liraglutide decreased HbA1c values by 0.7% in an Arab population when used in combination with other anti-diabetic agents [17]. In this study, the mean HbA1c values dropped by 0.9% using exenatide, which was not as efficient as with liraglutide, but which was consistent with a previous study that showed that liraglutide could decrease HbA1c more effectively than exenatide (-0.86% for liraglutide and -0.61% for exenatide) [18]. In another aspect, both liraglutide and exenatide could decrease body weight, reflecting by a reduced BMI by 1.33% and 1.12%, respectively. A previous study showed that there was no difference between liraglutide and exenatide regarding decreases of body weight [18]. These results are consistent between studies.

A 30-year simulated treatment with 1.2 mg of liraglutide and 10 μg of exenatide showed that liraglutide had an incremental cost effectiveness of -11,550 RMB/QALYs, which was superior to exenatide. The Bootstrap scatter plot showed that the dots were mainly concentrated in the right lower quadrant indicating that liraglutide treatment was an economic strategy.

In this study, there were 23.2% and 19.4% of patients surviving after 30 years in the liraglutide and exenatide groups, respectively. Survival dropped below 50% after 23 and 22 years of treatment with liraglutide and exenatide treatment, respectively. In addition, the differences in life expectancy and QALY were 0.471 years and 0.388 QALYs higher under liraglutide treatment than under exenatide, resulting in a incremental cost/effectiveness was -11,550 RMB/QALYs with liraglutide. Tzanetakos et al. [19] performed a CORE analysis and showed that over a life time treatment in a Greek setting, liraglutide vs. exenatide resulted in discounted life expectancy of 0.14 years and QALY of 0.16, resulting in a better cost-effectiveness of liraglutide. McDonell et al., [20] showed that the daily cost of two injections of exenatide was lower that one injection of liraglutide in Germany, the Netherlands, and the United Kingdom, but they did not perform a long-term CORE model and a previous study suggested that the source of data might influence the conclusions about the cost-effectiveness of exenatide vs. liraglutide [21]. DeKoven et al. [22] showed that although the predicted costs of liraglutide were higher than those of exenatide, a higher proportion of patients under liraglutide treatment might reach target HbA1c levels.

Some other studies examined the cost-effectiveness of liraglutide, but compared to other drugs than exenatide. Gao et al. [8] performed a 30-year model and showed that liraglutide 1.8 mg/day was associated with better life expectancy, QALY, and cost-effectiveness ratio compared to glimepiride. Another study by Roussel et al. [23] in France showed that liraglutide was more cost-effective than sitagliptin and glimepiride. Finally, a systematic review suggested that liraglutide might be the most cost-effective treatment for T2DM, but the authors warn that the results between studies are largely dependent upon the assumptions regarding the long-term benefits [24].

This study is not without limitations. 1) The sample size was relatively small and some of the measurements for the CORE model could be collected and supplemented by other sources, which might affect the accuracy of our findings. In addition, many studies used the CORE model for Asian populations [8,25–28], but the model has never been validated for Asian population. Additional studies with a larger sample size and longer follow-up are still necessary to confirm these findings. 2) The none-measurable costs such as depreciation charge for medical equipment, indirect medical costs, disease onset and death costs, and other intangible costs including pain and sadness were not included into the study. 3) The present study is based on epidemiological studies (UKPDS, Fei Minghan Heart Study, and DCCT), which may result in bias. Therefore, prospective data are needed to improve the epidemiological data for the validation of the effectiveness of the CORE model in China. 4) Drug cost and medical equipment depreciation costs also need to be analyzed in future studies. 5) Finally, further studies are needed to confirm the BMI results.

## Conclusions

In conclusion, the long-term effectiveness of liraglutide and exenatide was compared in this study, and it was shown that once-a-day injection of liraglutide was superior to twice-a-day injection of exenatide in terms of cost and effectiveness in Chinese patients with T2DM. It is the first report about the long-term effectiveness and cost-effectiveness of metformin combined with liraglutide or exenatide for T2DM based on the CORE diabetes model study based on Chinese population. Furthermore, the results based on a 52-week follow-up provide valuable information for clinicians when selecting a therapy.

## Supporting Information

S1 STROBE Checklist STROBE Statement—checklist of items that should be included in reports of observational studies. (DOC)

S1 Fig 30-year simulated HbA1c curve of diabetes patients. Blue is liraglutide and green is exenatide.
(DOCX)

S2 Fig 30-year simulated BMI curve of diabetes patients. Blue is liraglutide and green is exenatide.
(DOCX)

S1 Table Therapeutic effects after 52 weeks of treatment. (DOCX)

S2 Table Onset time of diabetic complications during the survival period of the patients. (DOCX)

S3 Table The cumulative rate of diabetic complications with the period preset to 40 years. (DOCX)

S4 Table The cumulative rate of diabetic complications with the period preset to 50 years. (DOCX)
