# Supplementary material for: Long-Term Effectiveness and Cost-Effectiveness of Metformin Combined with Liraglutide or Exenatide for Type 2 Diabetes Mellitus Based on the CORE Diabetes Model Study
Source: PLoS One. 2016 Jun 15;11(6):e0156393. doi: 10.1371/journal.pone.0156393 (PMC4909290; doi:10.1371/journal.pone.0156393)
Supplement: S1 Fig — Blue is liraglutide and green is exenatide. (DOCX) [file pone.0156393.s002.docx]

**S1 Fig. 30-year simulated HbA1c curve of diabetes patients.**


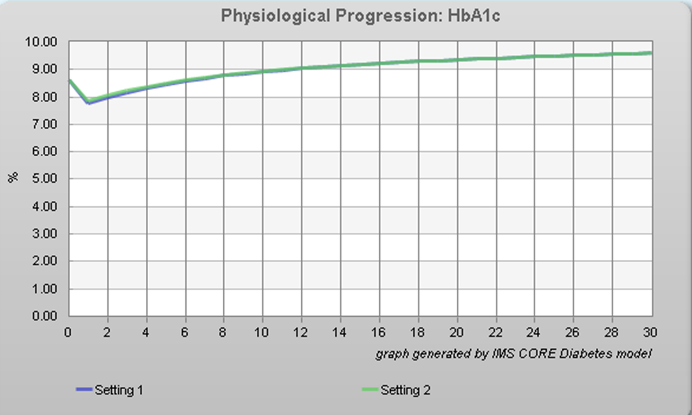


Blue is liraglutide and green is exenatide.
