# Supplementary material for: Long-Term Effectiveness and Cost-Effectiveness of Metformin Combined with Liraglutide or Exenatide for Type 2 Diabetes Mellitus Based on the CORE Diabetes Model Study
Source: PLoS One. 2016 Jun 15;11(6):e0156393. doi: 10.1371/journal.pone.0156393 (PMC4909290; doi:10.1371/journal.pone.0156393)
Supplement: S1 Table — (DOCX) [file pone.0156393.s004.docx]

**S1 Table.** **Therapeutic effects after 52 weeks of treatment.**

| Variables | Liraglutide | Exenatide |
| --- | --- | --- |
| HbA1c changes (%) | -1.1±1.3 | -0.9±0.8 |
| BMI changes (kg/m^2^) | -1.33±2.3 | -1.12±1.3 |
| Systolic changes (mmHg) | -2.7±17.2 | -1.9±15 |
| Total cholesterol changes (mmol·L^-1^) | -0.06±1.3 | -1.1±0.6 |
| High density lipoprotein changes (mmol·L^-1^) | 3.6±0.3 | -1.2±0.4 |
| Low density lipoprotein changes (mmol·L^-1^) | -0.28±1.1 | -0.30±1.0 |
| Triglyceride changes (mmol·L^-1^) | 1.3±0.6 | -2.8±1.9 |

Data are expressed as mean ± SD.
