# Supplementary material for: Long-Term Effectiveness and Cost-Effectiveness of Metformin Combined with Liraglutide or Exenatide for Type 2 Diabetes Mellitus Based on the CORE Diabetes Model Study
Source: PLoS One. 2016 Jun 15;11(6):e0156393. doi: 10.1371/journal.pone.0156393 (PMC4909290; doi:10.1371/journal.pone.0156393)
Supplement: S2 Table — (DOCX) [file pone.0156393.s005.docx]

**S2 Table.** **Onset time of diabetic complications during the survival period of the patients.**

| **Complications** | **Liraglutide(years)** | **Exenatide(years)** | **Changes(years)** |
| --- | --- | --- | --- |
| Any complications | 5.45 | 5.21 | 0.24 |
| Background retinopathy | 16.69 | 16.07 | 0.62 |
| Proliferative retinopathy | 20.06 | 19.24 | 0.82 |
| Microalbuminuria | 17.54 | 16.92 | 0.62 |
| Important proteinuria | 19.81 | 19.04 | 0.77 |
| End stage renal disease | 20.39 | 19.56 | 0.83 |
| Foot ulcer (first) | 17.47 | 16.83 | 0.64 |
| Amputation (first) | 19.67 | 18.89 | 0.78 |
| Neuropathy | 10.48 | 10.19 | 0.29 |
| Peripheral vascular disease | 18.53 | 17.76 | 0.77 |
| Congestive heart failure | 19.13 | 18.32 | 0.81 |
| Angina pectoris | 18.10 | 16.93 | 1.17 |
| Myocardial infarction | 19.26 | 18.14 | 1.12 |
| Stroke | 19.44 | 18.58 | 0.86 |
| Cataract | 18.48 | 17.79 | 0.69 |
| Macular edema | 17.12 | 16.48 | 0.64 |
| Severe visual impairment | 19.24 | 18.48 | 0.76 |
