# Supplementary material for: Long-Term Effectiveness and Cost-Effectiveness of Metformin Combined with Liraglutide or Exenatide for Type 2 Diabetes Mellitus Based on the CORE Diabetes Model Study
Source: PLoS One. 2016 Jun 15;11(6):e0156393. doi: 10.1371/journal.pone.0156393 (PMC4909290; doi:10.1371/journal.pone.0156393)
Supplement: S3 Table — (DOCX) [file pone.0156393.s006.docx]

**S3 Table. The cumulative rate of diabetic complications with the period preset to 40 years.**

| **Compilations** | | **Liraglutide(%)** | **Exenatide(%)** | **Changes(%)** |
| --- | --- | --- | --- | --- |
| Eye |  |  |  |  |
|  | Background retinopathy | 28.685 | 27.371 | 1.314 |
|  | Proliferative retinopathy | 0.686 | 0.613 | 0.073 |
|  | Severe visual impairment | 13.376 | 12.537 | 0.839 |
|  | Macular edema | 27.64 | 26.534 | 1.106 |
|  | Cataract | 14.176 | 13.698 | 0.478 |
| Kidney |  |  |  |  |
|  | Microalbuminuria | 28.881 | 27.348 | 1.533 |
|  | Large amount of proteinuria | 10.942 | 9.809 | 1.133 |
|  | End stage renal disease | 2.459 | 2.076 | 0.383 |
|  | Kidney related death | 1.89 | 1.616 | 0.274 |
| Foot |  |  |  |  |
|  | Foot ulcer (first) | 44.643 | 42.058 | 2.585 |
|  | Foot ulcer (repeated) | 66.273 | 61.135 | 5.138 |
|  | Amputation (first) | 14.221 | 13.113 | 1.108 |
|  | Amputation (multiple times) | 5.875 | 5.369 | 0.506 |
| Nervous system |  |  |  |  |
|  | Neuropathy | 70.815 | 68.414 | 2.401 |
| Blood vessel |  |  |  |  |
|  | Peripheral vascular disease | 22.566 | 22.014 | 0.552 |
|  | Congestive heart failure (disease) | 26.221 | 25.058 | 1.163 |
|  | Congestive heart failure (death) | 14.129 | 13.055 | 1.074 |
|  | Angina pectoris | 23.682 | 26.678 | -2.996 |
|  | Myocardial infarction (onset) | 36.006 | 43.378 | -7.372 |
|  | Myocardial infarction (death) | 28.374 | 34.125 | -5.751 |
|  | Stroke (onset) | 31.135 | 30.408 | 0.727 |
|  | Stroke (death) | 16.95 | 16.531 | 0.419 |
| Mild hypoglycemia event |  | 22.013 | 20.682 | 1.331 |
| Lactic acidosis |  | 17.041 | 16.012 | 1.029 |
