# Supplementary material for: Long-Term Effectiveness and Cost-Effectiveness of Metformin Combined with Liraglutide or Exenatide for Type 2 Diabetes Mellitus Based on the CORE Diabetes Model Study
Source: PLoS One. 2016 Jun 15;11(6):e0156393. doi: 10.1371/journal.pone.0156393 (PMC4909290; doi:10.1371/journal.pone.0156393)
Supplement: S4 Table — (DOCX) [file pone.0156393.s007.docx]

**S4 Table. The cumulative rate of diabetic complications with the period preset to 50 years.**

| **Compilations** | | **Liraglutide(%)** | **Exenatide(%)** | **Changes(%)** |
| --- | --- | --- | --- | --- |
| Eye |  |  |  |  |
|  | Background retinopathy | 28.986 | 27.55 | 1.436 |
|  | Proliferative retinopathy | 0.7 | 0.642 | 0.058 |
|  | Severe visual impairment | 13.625 | 12.706 | 0.919 |
|  | Macular edema | 27.883 | 26.655 | 1.228 |
|  | Cataract | 14.373 | 13.771 | 0.602 |
| Kidney |  |  |  |  |
|  | Microalbuminuria | 29.06 | 27.507 | 1.553 |
|  | Large amount of proteinuria | 10.942 | 10.022 | 0.92 |
|  | End stage renal disease | 2.479 | 2.097 | 0.382 |
|  | Kidney related death | 1.938 | 1.591 | 0.347 |
| Foot |  |  |  |  |
|  | Foot ulcer (first) | 44.85 | 42.51 | 2.34 |
|  | Foot ulcer (repeated) | 66.933 | 62.33 | 4.603 |
|  | Amputation (first) | 14.415 | 13.393 | 1.022 |
|  | Amputation (multiple times) | 6.028 | 5.543 | 0.485 |
| Nervous system |  |  |  |  |
|  | Neuropathy | 71.014 | 68.906 | 2.108 |
| Blood vessel |  |  |  |  |
|  | Peripheral vascular disease | 22.842 | 22.329 | 0.513 |
|  | Congestive heart failure (disease) | 26.634 | 25.584 | 1.05 |
|  | Congestive heart failure (death) | 14.715 | 13.628 | 1.087 |
|  | Angina pectoris | 23.986 | 26.95 | -2.964 |
|  | Myocardial infarction (onset) | 36.912 | 44.065 | -7.153 |
|  | Myocardial infarction (death) | 29.236 | 34.882 | -5.646 |
|  | Stroke (onset) | 32.027 | 30.887 | 1.14 |
|  | Stroke (death) | 17.731 | 16.871 | 0.86 |
| Mild hypoglycemia event |  | 22.189 | 20.865 | 1.324 |
| Lactic acidosis |  | 17.17 | 16.147 | 1.023 |
